# Focal Fat Infarction of the Falciform Ligament: Typical CT Appearance of a Rare Form of Intra-peritoneal Focal Fatty Infarction

**DOI:** 10.5334/jbsr.1793

**Published:** 2019-05-09

**Authors:** Louis Vanderschueren, Bruno Coulier

**Affiliations:** 1 St Luc Bouge (Namur), BE; 2 Clinique Saint-Luc, Bouge, BE

**Keywords:** Falciform ligament, Intra-abdominal focal fat infarction (IFFI), Abdomen, Computed tomography

## Case Reports

**Figure 1:**
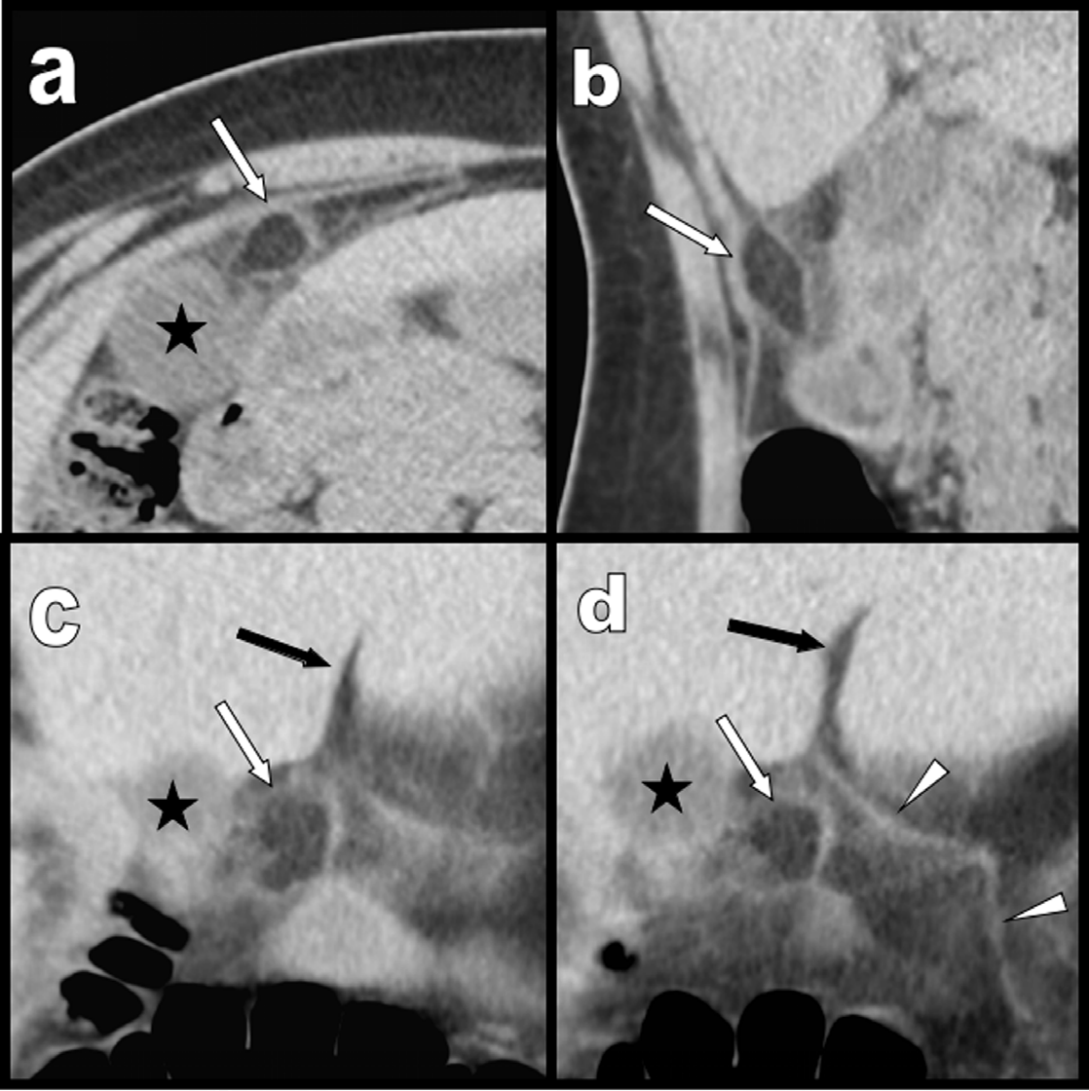
Case 1: transverse **(a)**, sagital **(b)** and coronal oblique **(c)** and **(d)** multiplanar reconstructions show a pre-hepatic area of fat stranding surrounding a 1,5 cm well demarcated inflammatory lipomatous process (white arrows) just under the hepatic fissure (black arrow) and in very close vicinity of the right edge of the ligamentum teres (white arrowheads). Black star = gallbladder.

**Figure 2:**
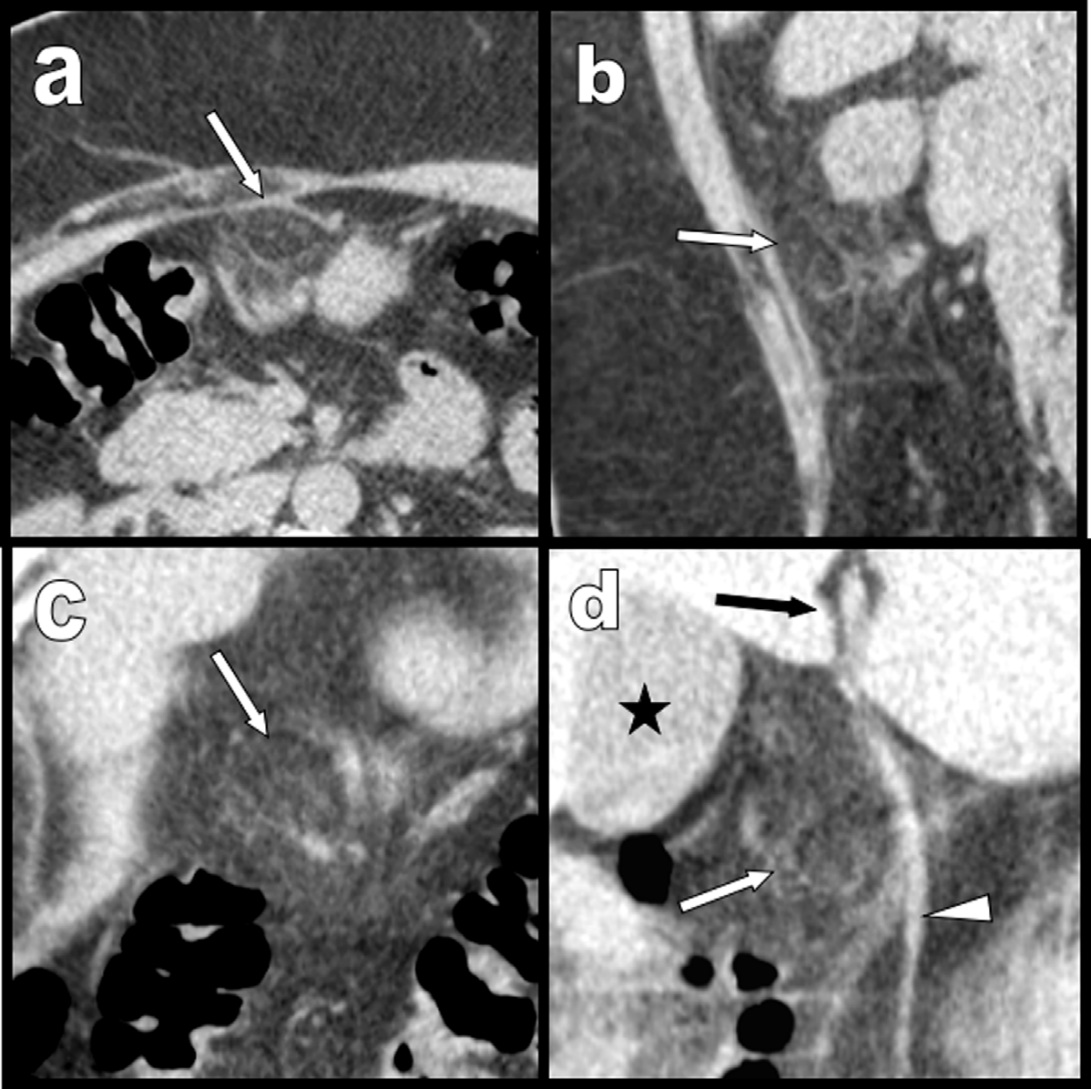
Case 2: similarly to case 1, transverse **(a)**, sagital **(b)** and coronal oblique **(c)** and **(d)** multiplanar reconstructions also show a pre-hepatic area of fat stranding surrounding a 1,5 cm inflammatory lipomatous mass (white arrows) just under the hepatic fissure (black arrow) and in very close vicinity of the ligamentum teres (white arrowheads). Black star = gallbladder. The background noise is more marked than in case one and is related to the obesity of the second patient.

In less than three months, two middle-aged women – 51 (Figure 1) and 53 (Figure 2) years old – were referred to our department with very similar complaints of acute pain in the right upper epigastric area. Both had epigastric defense, nausea, and moderate biological inflammatory syndrome. Emergency ultrasound excluded gallstones and/or cholecystitis in both but failed to provide a definite diagnosis. Complementary abdominal computed tomography (CT) showed localized pre-hepatic area of fat stranding, surrounding a 1.5 cm well-demarcated inflammatory lipomatous process (white arrows). These findings were precisely located beneath the projection of the hepatic fissure (black arrow), close to the inner side of the gallbladder (black star) and in very close vicinity of the right edge of the ligamentum teres (white arrowheads). These findings were typical of intra-abdominal focal fatty infarction (IFFI) involving an appendage of the falciform ligament. Both women were successfully treated conservatively.

## Comment

The typical radiological features of infarction and/or torsion of the greater omentum and epiploic appendages have been extensively reported during the last two decades. Similar imaging findings have also been more recently sporadically reported, including infarction and/or torsion of other peritoneum-covered lipomatous components of the gastrohepatic (lesser omentum), gastro-splenic, and falciform ligaments.

All these clinical entities have the same clinical acute presentation, etiology, radiological features, prognosis, and treatment. They only differ in their anatomical location or dimensions. The common denominator is fatty tissue necrosis, and for this reason the general term of intra-abdominal focal fatty infarction (IFFI) has been introduced.

IFFI of lipomatous appendage of the falciform ligament is by far the rarest type, first radiologically described in 2001 [1]. Up to now only 12 cases have been reported [2].

The double-layered peritoneal falciform ligament starts at the umbilicus and traces back to the liver at the level of the hepatic fissure separating the right and left anatomic hepatic lobes. It contains the ligamentum teres, the obliterated umbilical vein, and a variable quantity of extraperitoneal fat and/or fatty appendages.

IFFIs of the falciform ligament typically present with acute epigastric and/or right upper quadrant pain with or without constitutional symptoms and closely mimic other common gastroduodenal or bilio-pancreatic diseases comprising cholecystitis.

The sonographic diagnosis may reveal difficult in obese patients. It is merely useful to exclude cholecystitis.

CT classically demonstrates an area of fat density with a thin peripheral rim of hyper attenuation and with or without the classical “central dot” corresponding to the thrombosed vein. CT also clearly identify the vicinity of the ligamentum teres making unambiguous the diagnosis of IFFI of appendage of falciform ligament that allows a conservative treatment in the vast majority of cases.
